# Supplementary material for: Air pollution exposure associates with increased risk of neonatal jaundice
Source: Nat Commun. 2019 Aug 20;10:3741. doi: 10.1038/s41467-019-11387-3 (PMC6702167; doi:10.1038/s41467-019-11387-3)
Supplement: Supplementary file 1 — Supplementary Information [file 41467_2019_11387_MOESM1_ESM.docx]

**Supplementary Information**

**Air pollution exposure associates with increased risk of neonatal jaundice**


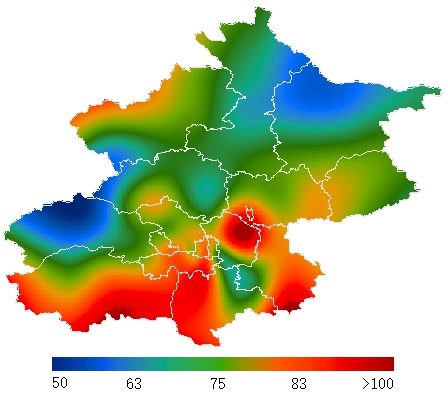

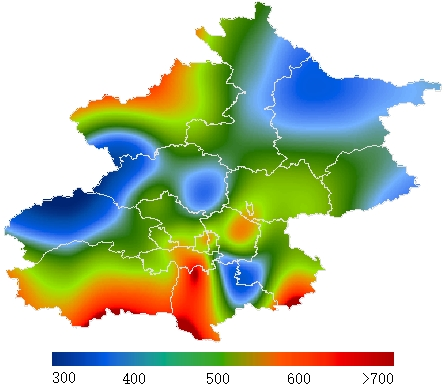

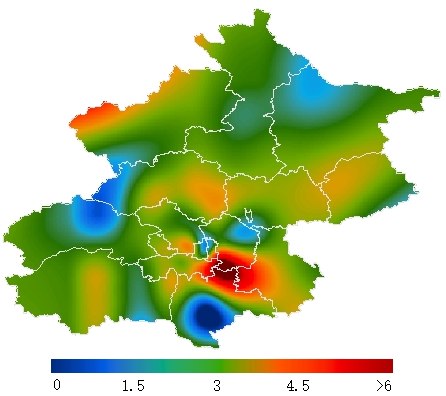


**a b c**

## Supplementary Figure 1. Spatial distribution of daily ambient PM_2.5_ pollution in Beijing over June 2014–May 2017. a, Average daily PM_2.5_ concentration (μg/m^3^) derived from the 34 air pollution monitoring stations. b, Maximum daily (μg/m^3^). c, Minimum daily (μg/m^3^). Note that the color scales differ between a, b and c.


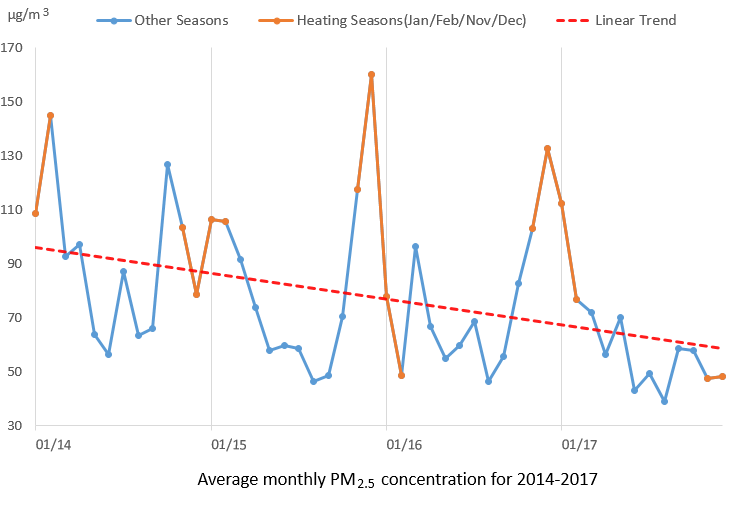


**a**


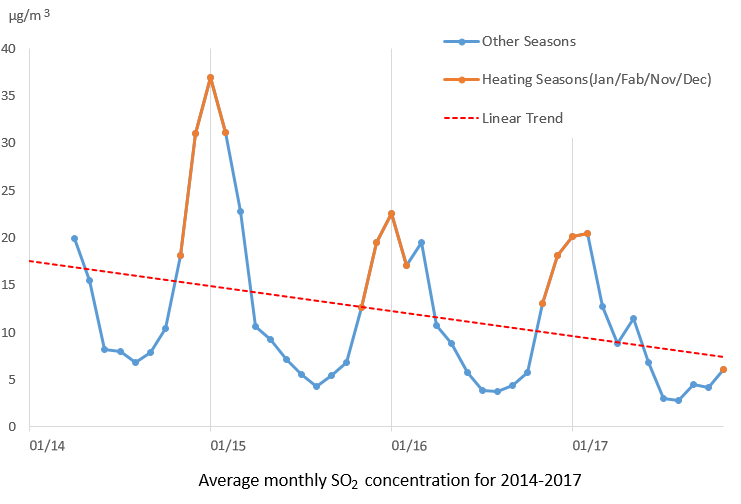


**b**


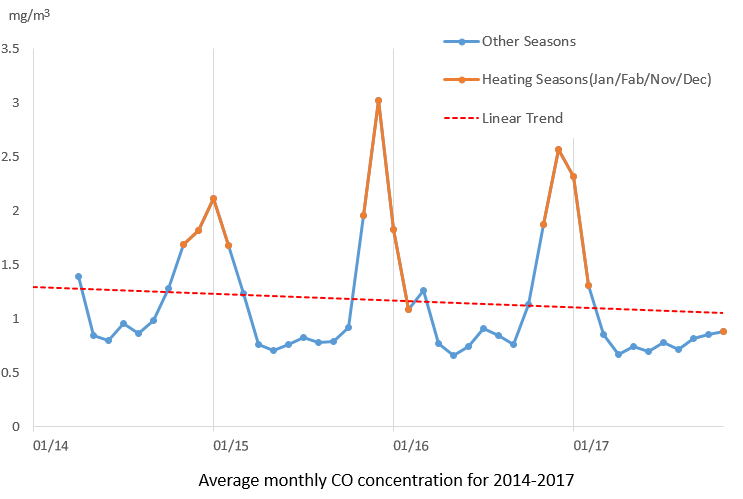


**c**


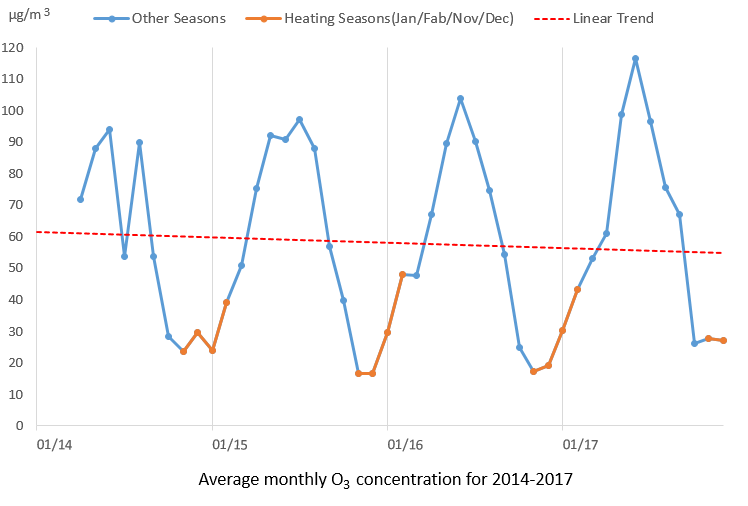


**d**


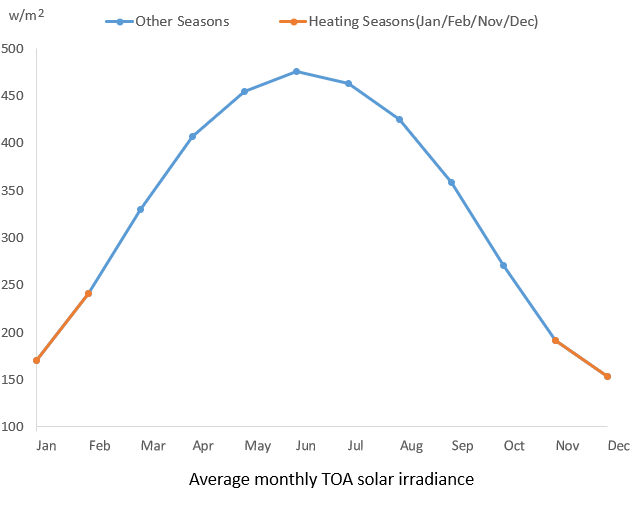


**e**


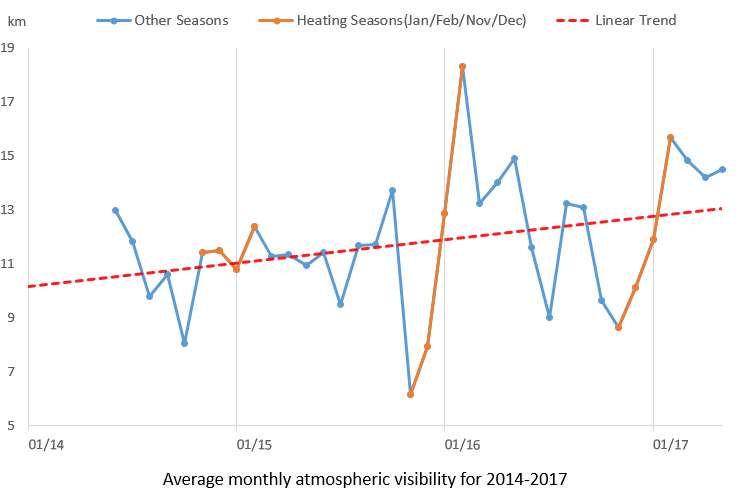


**f**

## Supplementary Figure 2. Time series for monthly mean air pollution exposure levels, TOA solar irradiance and atmospheric visibility. TOA irradiance does not change from one year to another, thus only the seasonal cycle is shown.

**a**

**b**

**c**

**Supplementary Figure 3. Partial response plots for peak bilirubin levels with respect to maternal pollution exposure.** Effects of neonatal pollution exposure concentration and time were controlled. The y-axis represents the marginal effects. The dashed lines represent 95% CI. The vertical lines adjacent to the x-axis represent the frequency of the data.

**a**

**b**

**c**

**Supplementary Figure 4. Partial response plots for peak bilirubin levels with respect to average pollution concentration.** The y-axis represents the marginal effects. The dashed lines represent 95% CI. The vertical lines adjacent to the *x*-axis represent the frequency of the data.

**a**

**b**

**c**

**Supplementary Figure 5. Partial response plots for peak bilirubin levels with respect to the time of neonatal exposure to air pollution.** The y-axis represents the marginal effects. The dashed lines represent 95% CI. The vertical lines adjacent to the *x*-axis represent the frequency of the data.

**Supplementary Figure 6. Relationship between PM_2.5_ concentration and atmospheric visibility.** The y-axis represents atmospheric visibility, and the *x*-axis denotes average PM_2.5_ concentrations.

## Supplementary Figure 7. The relationship between peak bilirubin levels in blood of 300 newborns and AQI. The AQI is below 100 at Level 1 and 2, which is designated as having relatively good air quality. The AQI exceeds 100 for Levels 3–6, which is designated as having relatively bad air quality.

## Supplementary Table 1. Recommended standards of neonatal jaundice intervention for full-term newborns of different birth days according to the Chinese clinical guideline of neonatal jaundice.

| Days after birth | TSB level (mg/dL) | |
| --- | --- | --- |
|  | Jaundice considering phototherapy | Jaundice requiring phototherapy |
| ≤ 1 | ≥ 6 | ≥ 9 |
| 2 | ≥ 9 | ≥ 12 |
| 3 | ≥ 12 | ≥ 15 |
| >3 | ≥ 15 | ≥ 17 |

## Supplementary Table 2. Correlations between different air pollution exposures as well as between air pollutants and atmospheric visibility based on daily mean air pollution data over June 2014–May 2017 obtained from 34 air pollution monitoring stations. PM_2.5_ was highly correlated with SO_2_ and CO, whereas O_3_ was negatively correlated with SO_2_ and CO. They reflected the air pollution mix on bad air quality days. There was the greatest negative correlation between PM_2.5_ exposure and atmospheric visibility, mainly because PM_2.5_ absorbs and scatters sunlight. We also note that SO_2_ and CO were negatively correlated with atmospheric visibility, and O_3_ was positively correlated with atmospheric visibility, largely reflecting the relationships between these pollutants and PM_2.5_.

|  | SO_2_ | O_3_ | CO | Visibility |
| --- | --- | --- | --- | --- |
| PM_2.5_ | 0.542 | -0.250 | 0.849 | -0.667 |
| SO_2_ |  | -0.366 | 0.635 | -0.376 |
| O_3_ |  |  | -0.398 | 0.074 |
| CO |  |  |  | -0.490 |

##

## Supplementary Table 3. Cohort characteristics and assessment results in the two groups. Statistically significant differences exist between the two groups (P < 0.05) in the occurrence of relatively bad air during the observation period. This suggests potential associations between these factors and incidence of neonatal jaundice.

| Group  Factor | | | Group I: non-jaundiced (n=11,724) | Group II: jaundiced (n=14,058) | P values |
| --- | --- | --- | --- | --- | --- |
| Mother age | | | 30.87±3.70 | 30.78±3.51 | 0.080 |
| Sex | | M | 5,910 (50.4%) | 7,440 (52.9%) | 0.120 |
|  | | F | 5,814 (49.6%) | 6,618 (47.1%) |  |
| Educational level | Junior high school or below | | 129 | 267 | 0.082 |
|  | Senior high school | | 348 | 483 | 0.095 |
|  | College or higher | | 11,247 | 13,308 | 0.127 |
| Occupation | Farmer | | 98 | 214 | 0.004 |
|  | Office worker | | 8,407 | 9,826 | 0.023 |
|  | Other | | 3,219 | 4,018 | 0.007 |
| Gestational age (week) | | | 39.14±1.24 | 39.14±1.13 | 0.632 |
| Birth weight (g) | | | 3,393.21±412.60 | 3,390.79±397.86 | 0.817 |
| Hypertension in pregnancy | | | 462 | 720 | 0.000 |
| Gestational diabetes | | | 1,410 | 1,644 | 0.411 |
| Fetal distress in uterus | | | 1,920 | 3,324 | 0.000 |
| Premature rupture of membranes | | | 2,910 | 3,444 | 0.550 |
| Cord around neck | | | 2,022 | 2,706 | 0.000 |
| Infection | | | 1,482 | 2,298 | 0.000 |
| Hypothyroidism | | | 468 | 684 | 0.001 |
| Anemia in pregnancy | | | 402 | 486 | 0.901 |
| Pollution exposure^*^ | | | 2,622 | 7,890 | 0.000 |

*Pollution exposure means exposure to air with AQI > 100 for more than 24 hours from birth to when peak bilirubin level is measured.

## Supplementary Table 4. Jaundiced newborns who were considered phototherapy or required phototherapy. For the 6,336 newborns who had the risk of clinically significant jaundice and required close monitoring and prompt treatments, this table lists the number of jaundiced newborns who were considered phototherapy (Those who should receive phototherapy usually received it. If they did not receive it, their bilirubin levels should be closely monitored. Once their bilirubin levels reached the degree of the required phototherapy, they must receive phototherapy) and required phototherapy (Those who were required to receive phototherapy actually received phototherapy).

| Days after birth | no. of Newborns (TSB level, mg/dL) | |
| --- | --- | --- |
|  | Jaundice considering phototherapy | Jaundice requiring phototherapy |
| ≤ 1 | 426 (≥ 6) | 736 (≥ 9) |
| 2 | 573 (≥ 9) | 934 (≥ 12) |
| 3 | 891 (≥ 12) | 1,152 (≥ 15) |
| >3 | 667 (≥ 15) | 957 (≥ 17) |
| Sum of newborns | 2,557 | 3,779 |

## Supplementary Table 5. Association between jaundice severity and concentrations of individual air pollutants. Concentrations of individual air pollutants (average and maximum values from the day of birth to the day before the peak bilirubin level was measured) for the close monitoring subgroup (Subgroup II) were higher than those in Subgroup I. The inter-group difference in mean concentration was statistically significant for PM_2.5_ and SO_2_; and the inter-group difference in maximum concentration was statistically significant for PM_2.5_, SO_2_ and CO. These results further supported that more severe jaundice tended to occur in more polluted air environments.

| Concentrations of air pollutants | First subgroup  (n=7,722) | Second subgroup  (n=6,336) | T value | P value |
| --- | --- | --- | --- | --- |
| Mean PM_2.5_ concentration (μg/m^3^) | 105.68±94.44 | 123.30±96.00 | -10.932 | 0.000 |
| Mean SO_2_ concentration (μg/m^3^) | 9.56±7.43 | 11.92±9.21 | -16.765 | 0.000 |
| Mean O_3_ concentration (μg/m^3^) | 32.33±23.66 | 30.52±21.31 | 1.930 | 0.054 |
| Mean CO concentration (mg/m^3^) | 1.55±1.40 | 1.64±1.18 | -4.164 | 0.004 |
| Max PM_2.5_ concentration (μg/m^3^) | 183.93±135.12 | 204.73±132.10 | -9.178 | 0.000 |
| Max SO_2_ concentration (μg/m^3^) | 19.88±15.04 | 23.90±16.10 | -15.242 | 0.000 |
| Max O_3_ concentration (μg/m^3^) | 82.66±57.40 | 79.91±56.17 | 1.162 | 0.246 |
| Max CO concentration (mg/m^3^) | 2.68±2.01 | 2.90±1.83 | -6.468 | 0.008 |

## Supplementary Table 6. Association between AQI and atmospheric visibility. In a polluted environment (AQI > 100), atmospheric visibility was much lower than that under less polluted environments (AQI < 100).

|  | AQI < 100 | AQI > 100 | T value | P value |
| --- | --- | --- | --- | --- |
| Mean visibility (km) | 13.18±7.89 | 6.90±5.26 | 19.083 | 0.000 |
| Max visibility (km) | 20.26±10.06 | 10.70±8.39 | 21.088 | 0.000 |
| Min visibility (km) | 5.81±5.30 | 3.27±2.37 | 9.990 | 0.000 |

## Supplementary Table 7. Association of the liver functions with air pollution concentrations. ALT and GGT levels were higher in newborns exposed to more serious air pollution (AQI > 100), although the difference was not statistically significant likely due to small sample size. Moreover, newborns exposed to poor air quality had a much higher AST level than those breathing cleaner air.

| Liver function (U/L) | AQI < 100 | AQI > 100 | T value | P value |
| --- | --- | --- | --- | --- |
| ALT | 15.20±6.72 | 15.63±5.24 | -0.342 | 0.733 |
| AST | 62.85±33.56 | 88.68±67.48 | -2.511 | 0.014 |
| GGT | 114.26±85.31 | 124.95±85.53 | -0.620 | 0.537 |

## Supplementary Table 8. Influences of air pollutants on bilirubin levels (controlling for atmospheric visibility and other confounding factors) performed by stratified analysis of daily TOA solar irradiance (95% CI). The TOA incident solar irradiance did not affect the relationship between air pollution exposure and the incidence of neonatal jaundice, by stratified analysis of TOA irradiance.

| Daily TOA irradiance (w/m^2^) | Pollutants | Exposure intervals | Estimated risk in peak bilirubin levels (mg/dL)* | P value |
| --- | --- | --- | --- | --- |
| 0-252.1 | PM_2.5_  (μg/m^3^) | (6.9, 10] | 0.848 (-0.574, 2.269) | 0.157 |
|  |  | (10, 35] | 0.076 (0.027, 0.125) | 0.003 |
|  |  | (35,75] | 0.029 (0.014,0.044) | 0.031 |
|  |  | (75, 200] | 0.009 (0.002,0.016) | 0.008 |
|  |  | > 200 | 0.010 (-0.008, 0.028) | 0.435 |
|  | SO_2_  (μg/m^3^) | (2, 5] | 0.082 (-0.157, 0.321) | 0.327 |
|  |  | (5, 10] | 0.028 (-0.113, 0.170) | 0.776 |
|  |  | (10, 15] | 0.095 (0.078,0.112) | < 0.001 |
|  |  | > 15 | 0.161 (0.070, 0.252) | < 0.001 |
|  | CO (mg/m^3^) | (0.3, 3.5) | 0.351 (0.305, 0.379) | 0.005 |
| 252.1-283.8 | PM_2.5_  (μg/m^3^) | (8.3, 10] | 0.848 (-0.574, 2.269) | 0.157 |
|  |  | (10, 35] | 0.076 (0.027, 0.125) | 0.003 |
|  |  | (35,75] | 0.029 (0.014,0.044) | 0.031 |
|  |  | (75, 200] | 0.009 (0.002,0.016) | 0.008 |
|  |  | > 200 | 0.010 (-0.008, 0.028) | 0.435 |
|  | SO_2_  (μg/m^3^) | (2, 5] | 0.082 (-0.157, 0.321) | 0.327 |
|  |  | (5, 10] | 0.028 (-0.113, 0.170) | 0.776 |
|  |  | (10, 15] | 0.095 (0.078,0.112) | < 0.001 |
|  |  | > 15 | 0.162 (0.071, 0.253) | < 0.001 |
|  | CO (mg/m^3^) | (0.3, 2.8) | 0.351 (0.305, 0.379) | 0.005 |
| 283.8-313.2 | PM_2.5_  (μg/m^3^) | (8, 10] | 0.848 (-0.574, 2.269) | 0.157 |
|  |  | (10, 35] | 0.076 (0.027, 0.125) | 0.003 |
|  |  | (35,75] | 0.029 (0.014, 0.044) | 0.031 |
|  |  | (75, 200] | 0.009 (0.002, 0.016) | 0.008 |
|  |  | >200 | 0.010 (-0.008, 0.028) | 0.435 |
|  | SO_2_  (μg/m^3^) | (2, 5] | 0.082 (-0.157, 0.321) | 0.327 |
|  |  | (5, 10] | 0.028 (-0.113, 0.170) | 0.776 |
|  |  | (10, 15] | 0.095 (0.078,0.112) | < 0.001 |
|  |  | > 15 | 0.161 (0.070, 0.252) | < 0.001 |
|  | CO (mg/m^3^) | (0.25, 2.6) | 0.351 (0.305, 0.379) | 0.005 |
| > 313.2 | PM_2.5_  (μg/m^3^) | (7.4, 10] | 0.848 (-0.574, 2.269) | 0.157 |
|  |  | (10, 35] | 0.076 (0.027, 0.125) | 0.003 |
|  |  | (35,75] | 0.029 (0.014, 0.044) | 0.031 |
|  |  | (75, 184.4] | 0.009 (0.002, 0.016) | 0.008 |
|  | SO_2_  (μg/m^3^) | (2, 5] | 0.082 (-0.157, 0.321) | 0.327 |
|  |  | (5, 10] | 0.027 (-0.114, 0.169) | 0.776 |
|  |  | (10, 14.1] | 0.095 (0.078,0.112) | ﹤0.001 |
|  | CO (mg/m^3^) | (0.3, 1.8) | 0.353 (0.316, 0.390) | 0.005 |

* on the basis of an increase of 1.0 μg/m^3^ in exposure to PM_2.5_, an increase of 1.0 μg/m^3^ in exposure to SO_2_, or an increase of 1.0 mg/m^3^ in exposure to CO.

## Supplementary Table 9. Influences of air pollutants on bilirubin levels performed by stratified analysis of atmospheric visibility. The association between air pollution exposure and jaundice is similar at different levels of visibility.

| Atmospheric visibility (km) | Pollutants | Exposure intervals | Estimated risk in peak bilirubin levels (mg/dL)* | P value |
| --- | --- | --- | --- | --- |
| < 4.7 | PM_2.5_  (μg/m^3^) | (75, 200] | 0.009 (0.002, 0.016) | 0.008 |
|  |  | >200 | 0.01 (-0.008, 0.028) | 0.631 |
|  | SO_2_  (μg/m^3^) | (3.8, 5] | 0.082 (-0.157, 0.321) | 0.256 |
|  |  | (5, 10] | 0.027 (-0.114, 0.169) | 0.776 |
|  |  | (10, 15] | 0.093 (0.076, 0.11) | < 0.001 |
|  |  | > 15 | 0.164 (0.073, 0.255) | < 0.001 |
|  | CO (mg/m^3^) | (1, 3.5) | 0.352(0.315, 0.389) | 0.005 |
| 4.7-8.2 | PM_2.5_  (μg/m^3^) | (41.6,75] | 0.029 (0.014, 0.044) | 0.031 |
|  |  | (75, 200] | 0.008 (0.001, 0.015) | 0.008 |
|  |  | > 200 | 0.011 (-0.007, 0.029) | 0.532 |
|  | SO_2_  (μg/m^3^) | [2, 5] | 0.082 (-0.157, 0.321) | 0.327 |
|  |  | (5, 10] | 0.032 (-0.109, 0.174) | 0.756 |
|  |  | (10, 15] | 0.095 (0.078, 0.112) | < 0.001 |
|  |  | > 15 | 0.162 (0.071, 0.253) | < 0.001 |
|  | CO (mg/m^3^) | (0.8, 3.5) | 0.350 (0.313-0.387) | 0.005 |
| 8.2-15.9 | PM_2.5_  (μg/m^3^) | (7.2, 10] | 0.894 (-0.528, 2.315) | 0.168 |
|  |  | (10, 35] | 0.071 (0.022, 0.12) | 0.003 |
|  |  | (35,75] | 0.03 (0.017, 0.047) | 0.031 |
|  |  | (75, 88.4] | 0.008 (0.001, 0.015) | 0.008 |
|  | SO_2_  (μg/m^3^) | (2, 5] | 0.082 (-0.157, 0.321) | 0.348 |
|  |  | (5, 10] | 0.027 (-0.114, 0.169) | 0.876 |
|  |  | (10, 15] | 0.092 (0.075, 0.109) | < 0.001 |
|  |  | > 15 | 0.162 (0.071, 0.253) | < 0.001 |
|  | CO | (0.3, 1.5) | 0.350 (0.313, 0.387) | 0.005 |
| >15.9 | PM_2.5_  (μg/m^3^) | (6.9, 10] | 0.846 (-0.576, 2.267) | 0.164 |
|  |  | (10, 35] | 0.078 (0.029, 0.127) | 0.003 |
|  |  | (35,75] | 0.027 (0.012, 0.042) | 0.031 |
|  |  | (75, 114.4] | 0.010 (0.003, 0.017) | 0.008 |
|  | SO_2_  (μg/m^3^) | (2, 5] | 0.082 (-0.157, 0.321) | 0.298 |
|  |  | (5, 10.3] | 0.031 (-0.11, 0.173) | 0.668 |
|  | CO (mg/m^3^) | (0.25, 0.9) | 0.352(0.315, 0.389) | 0.008 |

* on the basis of an increase of 1.0 μg/m^3^ in exposure to PM_2.5_, an increase of 1.0 μg/m^3^ in exposure to SO_2_, or an increase of 1.0 mg/m^3^ in exposure to CO.

To clarify the analyses by TOA and visibility, we added the following overall linear model for the entire exposure range, in addition to the presented exposure intervals.

*Y*=ε+α*Vis*+β*Irra*  (1)

where *Y* is the bilirubin level. *ε* is the intercept. α and β are parameters. *Vis* is atmospheric visibility. *Irra* is daily TOA irradiance.

Based on Equation (1), we analyzed the data and presented the results as shown in Supplementary Tables 10-11.

## Supplementary Table 10. Effects of daily TOA irradiance on bilirubin levels using Equation (1). The TOA irradiance had a weak negative correlation with bilirubin levels. As the value of the TOA irradiance enhanced, bilirubin levels decreased very slightly. Consistent with the results in Supplementary Table 8, the TOA irradiance had little impacts on the incidence of neonatal jaundice.

| Pollutants | Exposure intervals | Daily TOA irradiance (w/m^2^) | Estimated risk in peak bilirubin levels (mg/dL)* | P value |
| --- | --- | --- | --- | --- |
| PM_2.5_ (μg/m^3^) | (6.9, 350.4] | [207.6, 395.7] | -0.006 (-0.007, -0.004) | <0.001 |
| SO_2_  (μg/m^3^) | (2, 24.6] | [207.6, 395.7] | -0.006 (-0.007, -0.004) | <0.001 |
| CO (mg/m^3^) | (0.25, 3.5) | [207.6, 395.7] | -0.006 (-0.007, -0.004) | <0.001 |

* on the basis of an increase of 1.0 w/m^2^ in Daily TOA irradiance.

## Supplementary Table 11. Effects of atmospheric visibility on bilirubin levels using Equation (1). Atmospheric visibility was not statistically significantly associated with the increase in bilirubin levels (P>0.05). Thus, it indicates that bilirubin levels did not change with different levels of atmospheric visibility, consistent with the results listed in Supplementary Table 9.

| Pollutants | Exposure intervals | Atmospheric visibility (km) | Estimated risk in peak bilirubin levels (mg/dL) | P value |
| --- | --- | --- | --- | --- |
| PM_2.5_ (μg/m^3^) | (6.9, 350.4] | [1.1, 29.9] | 0.005 (-0.01, 0.021) | 0.502 |
| SO_2_  (μg/m^3^) | (2, 24.6] | [1.1, 29.9] | 0.005 (-0.01, 0.021) | 0.502 |
| CO (mg/m^3^) | (0.25, 3.5) | [1.1, 29.9] | 0.005 (-0.01, 0.021) | 0.502 |
